# Editorial: Vibrationally-Mediated Chemical Dynamics

**DOI:** 10.3389/fchem.2021.681457

**Published:** 2021-04-20

**Authors:** Jacob C. Dean, Doran I. G. Bennett, Michael Staniforth, Margherita Maiuri

**Affiliations:** ^1^Department of Physical Science, Southern Utah University, Cedar City, UT, United States; ^2^Department of Chemistry, Southern Methodist University, Dallas, TX, United States; ^3^Department of Chemistry, University of Warwick, Coventry, United Kingdom; ^4^Dipartimento di Fisica, Politecnico di Milano, Milano, Italy

**Keywords:** spectroscopy, density functional theory, conical intersections, vibronic coupling, isomerization

Nuclear motion, or molecular vibrations, mediate essentially all chemical dynamics and reactions. A rich interplay between electronic and nuclear degrees-of-freedom manifests in contexts such as light-induced chemical reactions (photochemistry), electron/charge transfer, and ultrafast energy dissipation. In these contexts, the close connection between chemical structure and molecular vibrations has inspired studies which navigate the *structure*-*vibration*-*function* relationship imprinted into the corresponding dynamics. Viewed in this way, molecular vibrations can act as a template for prescribing a particular function to a chemical structure, with the common goal being to design structural motifs which can predictably give rise to a target function in applications of energy capture and conversion, drug discovery, and quantum computing just to name a few. It is our privilege to introduce this Research Topic issue of *Frontiers in Chemistry* which includes a set of 8 articles which collectively journey into this frontier.

The presence of UV photons in the solar spectrum, with energy sufficient to break chemical bonds, obliges all terrestrial organisms to develop UV sunscreens. Importantly, an efficient UV-absorber does not by itself necessarily constitute an effective sunscreen. An ideal candidate must have strong absorptions in the UVA/UVB spectrum while being photostable and biologically inert. Horbury et al. present an investigation on the excited state behavior and screening efficacy of a sinapate-based UV sunscreen inspired by plants. The authors cleverly design a dimerized form of ethyl sinapate to successfully extend the absorption range of the sunscreen while hoping to retain the photostability of its precursor. Indeed, the candidate successfully relaxes back to its ground state with high yield, however under prolonged UV exposure additional photochemistry takes place. The work simultaneously reveals the importance of ultrafast and “ultraslow” timescales in the design of new sunscreens. In the paper presented by Whittock et al. the mycosporine family of UV sunscreens inspired from cyanobacteria, fungi, and algae were evaluated with ultrafast UV pump-IR probe spectroscopy in tandem with an impressive computational analysis. The authors quantified the ground state recovery following two relaxation events through conical intersections (CIs), indicating favorable dynamics for photostability. To accomplish this through probed IR signatures, the authors used density functional theory calculations of the S_0_ and S_1_ structures where explicit solvation was required due to a sensitivity of the vibrations to the solvent environment.

Biliproteins, found in a vast array of organisms, are unique in their use of linear tetrapyrrole pigments (bilins) for both light-harvesting/energy transfer and photoswitching. The two biological contexts call for different criteria for success: the former requires the pigment to remain rigid to avoid quenching, and the latter demands torsional isomerization for signaling. Staheli et al. characterized this behavior through a bottom-up approach by analyzing the spectroscopic/photophysical properties of the two dipyrrole halves of the bilin framework.

They found that already at the subunit level, the structures garner large absorptivities rivaling chlorophyll, with absorption regions highly sensitive to structure. Without a protein scaffolding however, the dipyrroles are susceptible to inter-ring torsion leading to facile internal conversion on ultrafast timescales. It was found that the local protein environment *in vivo* serves to then restrict or confer this behavior in a controllable way highlighting the interplay of “built-in” intramolecular dynamics and intermolecular interactions.

As displayed in these experimental works, the pervasiveness of conical intersections along “special” nuclear coordinates in steering ultrafast dynamics is an important facet to functional design. Therefore, screening the potential energy topology of photo-active target molecules is a crucial step to drug design in new light-induced therapies. Léger et al. report results from high-level multireference electronic structure calculations applied to a candidate for light-activated cancer treatment. The authors map out the potential energy structure leading to the active biradical form of a cyclopropenone precursor, successfully describing all steps and proving a Norrish type-I reaction mechanism through singlet states. Furthermore, calculations of spectral properties of derivatives of the cyclopropenone containing enediyne revealed potentially more effective compounds. This work demonstrates the power of quantum chemistry in informing targeted drug synthesis. Similarly, Cui et al. have used density functional theory to compare photostability and light-activated dynamics in an unnatural DNA base pair (ZP) along the proton transfer coordinates binding the pair, against their natural DNA counterparts. The authors find a notable difference in the potential energy landscapes along the primary proton transfer coordinates of the ZP pair relative to its GC natural equivalent. Importantly, a CI exists in the GC pair which allows ultrafast recovery of the ground state, while no such CI exists in the ZP pair, suggesting a reduced photostability in ZP following UV absorption.

Following a milestone work on the comprehensive description of the *Q*_x_/*Q*_y_ absorption region of chlorophyll-a (Chl-a), Reimers et al. present a holistic paper describing the asymmetry observed between high-resolution Chl-a absorption and fluorescence spectra. The authors combine low temperature (4.5 K) fluorescence line narrowing experiments in multiple solvents with spectral fitting to recover the vibronic properties of various active vibrations of Chl-a. In doing so, the authors present a detailed description of the vibrational content of Chl-a fluorescence spectra. They determine that solvent-induced differences in intensity are a result of Duschinsky mixing of certain modes between the ground and *Q*_y_ states. With the aid of DFT, the extent of mode-mixing was fully characterized and shown to cause absorption/fluorescence asymmetry. The authors highlight the importance of this result in modeling quantum dynamics of Chl-containing light-harvesting complexes.

Finally, vibronic coupling in the excited state manifold of multichromophoric systems has been implicated in facilitating and, at times, steering excited-state dynamics.  Sláma et al. demonstrate the utility of modeling donor-acceptor systems with a true vibronic model which treats the electronic and vibrational components equally—an approach that is theoretically practical in all electronic coupling regimes including the so-called intermediate regime where the timescales of both are similar. The authors apply the model to novel *peri*-arylene dyads in two limiting cases—one incorporating parallel transition dipole moments and another orthogonal—successfully reproducing steady-state spectra and population dynamics for both. The authors explain the discrepancy between the zero coupling of the orthogonal dyad and the fast energy transfer measured experimentally by demonstrating that anharmonicity in a multitude of low-frequency modes leads to a cooperative enhancement of electronic coupling through deviations of the nuclear displacements. The results of this work neatly express the utility of the vibronic treatment of molecular states, as well as statistical sampling at finite temperatures using molecular dynamics. Dunnett and Chin on the other hand, report a novel alternative where dissipative quantum dynamics are simulated at a finite temperature using 0 K wave functions. Armed with this novel method, the authors test and successfully simulate several model Hamiltonians, including a vibronic-tunneling system representative of electron transfer. In the process, the authors highlight numerical challenges but ultimately offer a promising vision for a new approach that uses wave function based calculations to simulate finite temperature systems.

## Author Contributions

JD, DB, and MS drafted, reviewed, and completed the manuscript for submission. All authors provided intellectual contributions to the work.

## Conflict of Interest

The authors declare that the research was conducted in the absence of any commercial or financial relationships that could be construed as a potential conflict of interest.

